# Ultrasound-guided modified versus conventional serratus anterior plane block as a preemptive analgesic for unilateral video-assisted thoracoscopic surgery

**DOI:** 10.1186/s12871-025-03314-5

**Published:** 2025-08-29

**Authors:** Shereen E. Abd Ellatif, Asmaa M. Galal Eldin, Ehab Sabry Ali, Heba M. Fathi

**Affiliations:** 1 Department of Anaesthesia, Intensive Care and Pain Management, Faculty of Medicine, Zagazig University, Zagazig, Egypt; 2 Department of Cardiothoracic Surgery, Faculty of Medicine, Zagazig University, Zagazig, Egypt

**Keywords:** Analgesia, Fascial plane block, Video-assisted thoracoscopic surgery, Regional anaesthesia, Serratus anterior plane block, Thoracic surgery.

## Abstract

**Purpose:**

Various approaches to serratus anterior plane (SAP) block have been discussed in the literature. The present study aimed to compare the analgesic efficacy and postoperative pulmonary function recovery of modified serratus anterior plane block (MSAP) and conventional serratus anterior plane block (CSAP) in patients undergoing video-assisted thoracoscopic surgery (VATS).

**Methods:**

A total of 99 patients who underwent thoracoscopic surgery were randomly divided into three equal groups: a control group (C group) that received no block, a CSAP group that received preoperative conventional serratus anterior plane block, and an MSAP group that received preoperative modified serratus anterior plane block. The primary outcome was the first 24-hour tramadol consumption. The secondary outcomes were first-time-to-rescue analgesia, postoperative visual analogue score (VAS), postoperative respiratory function, and perioperative hemodynamics.

**Results:**

The lowest tramadol consumption and longest time to first rescue analgesia were observed in the MSAP group. The postoperative VAS score at 2, 4, and 6 h was significantly greater in the control group, but it was comparable between the two block groups. At 8 h, the VAS score was the lowest in the MSAP group. At 12, 16, and 24 h, the VAS scores were comparable among the three groups. The MSAP group presented the best respiratory function during the first postoperative 8 h. Heart rate and mean arterial blood pressure were similar in both block groups but were greater in the control group during the intraoperative period.

**Conclusion:**

The modified serratus anterior plane block is more efficient than the conventional serratus anterior plane block at reducing opioid consumption, prolonging rescue analgesic time, and improving pulmonary function recovery in patients undergoing video-assisted thoracoscopic surgery.

**Trial registration:**

This clinical trial was approved by the Zagazig University Institutional Review Board (IRB #10060/30-10-2022), was first submitted to clinical trials.gov on 1/12/2022 and was subsequently registered retrospectively on 22/12/2022 (NCT05661253). The first research participant was enrolled on 2/12/2022.

**Supplementary Information:**

The online version contains supplementary material available at 10.1186/s12871-025-03314-5.

## Introduction

Patients who undergo thoracic surgery may experience significant postoperative pain [1]. The advent of video-assisted thoracoscopic surgery (VATS) has revolutionized the field, quickly increased in popularity and became the standard approach for thoracic surgery [2, 3]. Many operations, such as decortication, wedge resection, and sympathectomy, can be conducted via video imaging via endoscopic instruments in the thoracic cavity [4, 5]. Even with smaller incisions, no rib retraction, and minimal tissue injury, acute and chronic pain management remains a significant clinical challenge with VATS. This pain is both somatic and visceral in origin [6, 7].

Furthermore, ineffective management of postsurgical pain may increase the incidence of postoperative pulmonary complications (PPCs), such as hypoxia, atelectasis and chronic postsurgical pain, which can hinder early recovery. Many analgesic options have been suggested, and each technique has its own advantages and disadvantages. These methods include systemic analgesics, thoracic epidural block (TEB), thoracic paravertebral block (PVB), local anaesthetic infiltration of the wound and multilevel intercostal nerve block [8–10].

The conventional serratus anterior plane (CSAP) block is a regional anaesthesia technique in which local anaesthetics (LAs) are injected into the interfacial plane at the level of the fifth rib in the mid-axillary line, either superficially or deeply to the serratus anterior muscle [11]. CSAP provides effective hemithoracic analgesia by blocking the lateral branches of the intercostal nerves, usually between the T2-T9 levels and the long thoracic nerve of the bell supplying the serratus anterior muscle [12], including incisions made for VATS procedures and chest tube insertion sites on the anterolateral chest wall [13].

A further modification to the CSAP block was described by Khemka & Chakraborly, where the LAs are injected between the serratus anterior and latissimus dorsi (LD) muscles while the patient is in the lateral position. This block provides excellent postoperative analgesia in breast reconstruction surgery via the latissimus dorsi flap after mastectomies [14].

The aim of the current study was to compare the analgesic effects of the CSAP block with those of the modified serratus anterior plane (MSAP) block in patients undergoing VATS.

## Patients & methods

This prospective randomized controlled clinical trial was conducted at the Department of Anaesthesia, Intensive Care, and Pain Management at Zagazig University Hospitals. The study was approved by the Zagazig University Institutional Review Board (IRB #10060/30-10-2022) and was registered on clinicaltrials.gov (NCT05661253). The study was carried out according to the regulations and guidelines of Helsinki [15] and in adherence with CONSORT guidelines (http://www.consort-statement.org).

The study included patients scheduled for elective unilateral VATS under general anaesthesia. The participants were both male and female, aged between 21 and 70 years, and had a body mass index between 18 and 30 kg/m^2^. These patients were classified as American Society of Anaesthesiology physical status I or II. Written informed consent was obtained from all participants for their participation in the current study and for the publication of scientific findings.

The exclusion criteria included patients with a history of allergy to LA agents used in this study; skin lesions at the needle insertion site; a history of chronic pain and receiving analgesics; sepsis; significant renal, cardiac or liver diseases; bleeding disorders; anticoagulant therapy; uncooperative patients; or psychiatric disorders. The patients reserved the right to withdraw from the study at any time without any detrimental effects on their treatment plan.

The patients were recruited at the preanaesthesia clinic. Computer-generated random numbers were used to divide patients equally into three study groups, The patient was allocated according to these numbers, which were placed in sealed concealed envelopes that were opened on the day of surgery by an independent anaesthesiologist.

The control group (C group) included 33 patients who underwent surgery under general anaesthesia without preoperative nerve block.

CSAP group: (*n* = 33): patients who received a CSAP block with 25 ml of 0.25% bupivacaine followed by induction of general anaesthesia.

MSAP group: (*n* = 33): patients received MSAP block with a volume of 25 ml of 0.25% bupivacaine followed by induction of general anaesthesia.

### Preoperative Preparation

Preoperative visits for all participating patients were conducted, during which we discussed the aim and endpoints of the study, explained the advantages and potential side effects of the strategy and obtained written informed consent. During these visits, medical history, including previous operations and anaesthesia, was obtained, and physical examination, with a focus on documenting vital signs and chest and cardiac conditions, was performed to exclude contraindications. All patients were investigated by complete blood count, serum blood sugar level, coagulation profile, and liver and kidney function tests.

All patients were taught to quantify postoperative pain via a visual analogue scale ranging from 0 to 10 points, where 0 indicated no pain and 10 indicated the worst pain [16]. Finally, patients were instructed to fast for 6 h from solid meals and 2 h from clear liquids before the procedure.

### Intraoperative:

Upon arriving at the operating room, intravenous access via a cannula was secured. Patients were preloaded with 8–10 ml/kg Ringer’s lactate solution to compensate overnight fasting losses. Standard monitors, including electrocardiograms, non-invasive blood pressure monitoring, and pulse oximetry, were connected, and baseline parameters were measured and recorded.

The hand-held spirometer was used to measure the preoperative baseline forced vital capacity (FVC) and forced expiratory volume in one second (FEV1) after the patients had been trained to sit, take as deep a breath as possible, hold it for a few seconds, and then quickly and forcefully exhale into the mouthpiece until no more air came out. After three attempts on average, the values were recorded.

All patients received 0.05 mg/kg midazolam as premedication and supplemental oxygen via a simple face mask.

**Fig. 1:**
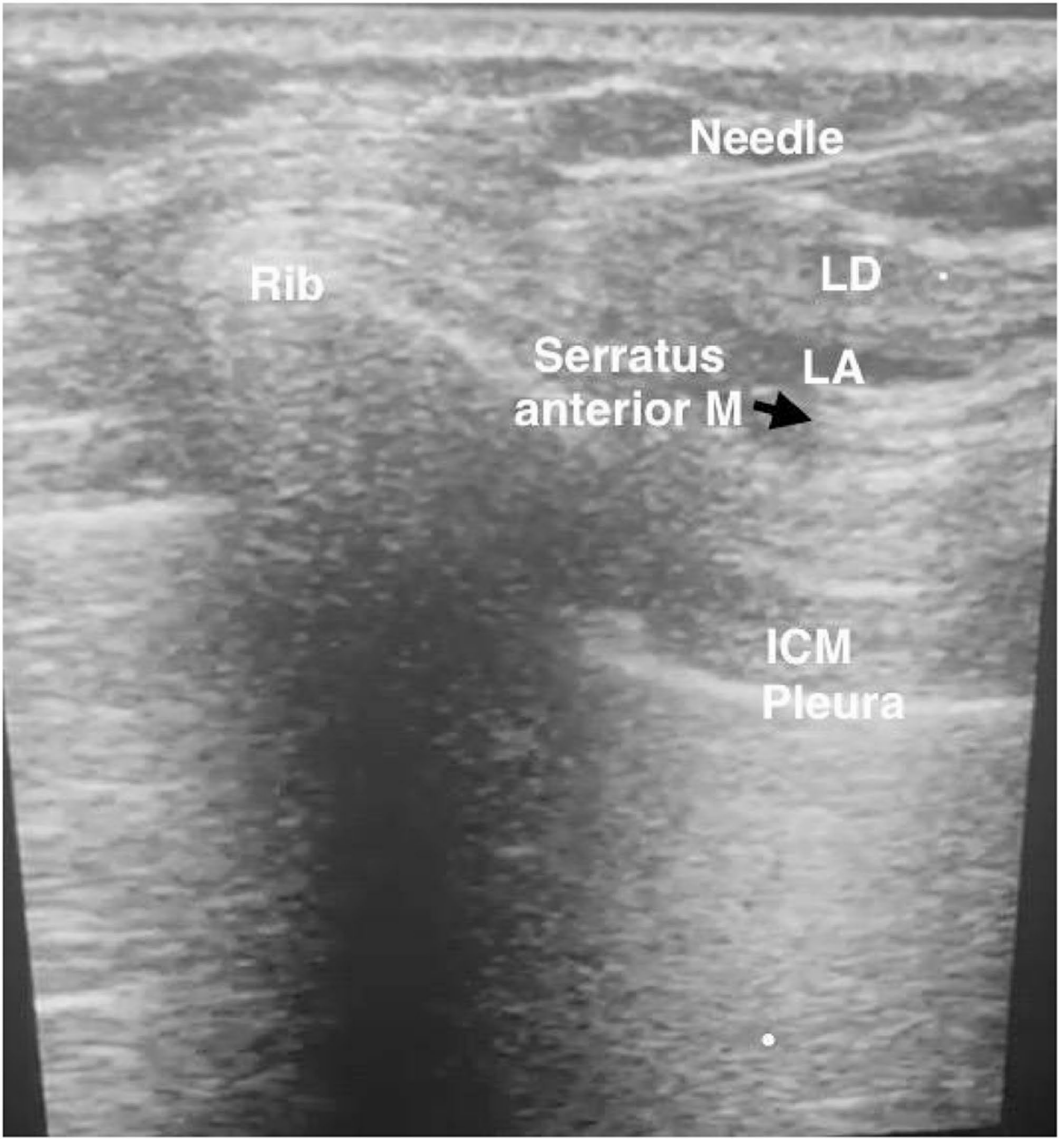
Sonographic image of the superficial serratus anterior plane block. LD: latissimus dorsi muscle, ICM: intercostal muscle, LA: local anesthetic spread between the serratus, and LD muscles

- ▪Technique of the CSAP block [11]:

Patients were placed in a supine position. After skin sterilization and draping, a high-frequency linear ultrasound probe (Siemens Medical Solutions, Inc., Mountain View, CA 94043, USA) was placed over the midclavicular region in the sagittal plane. The second rib was identified at the level of the axillary artery. The probe was moved inferiorly and laterally to locate the fifth rib in the mid-axillary line. At this point, the LD muscle was superficial and posterior, and the serratus anterior (SA) muscle was deep and inferior. A 22-gauge, 80 mm needle (Stimuplex D, B-Braun, Germany) was inserted in an anterior to posterior direction, in plane with the ultrasound probe, until the tip was positioned above the SA muscle. Negative aspiration confirmed the absence of blood, and 1 ml of normal saline was injected to visualize hydrodissection and verify needle tip placement. Subsequently, 25 ml of 0.25% bupivacaine was injected superficially into the serratus anterior muscle (Fig. 1).

**Fig. 2:**
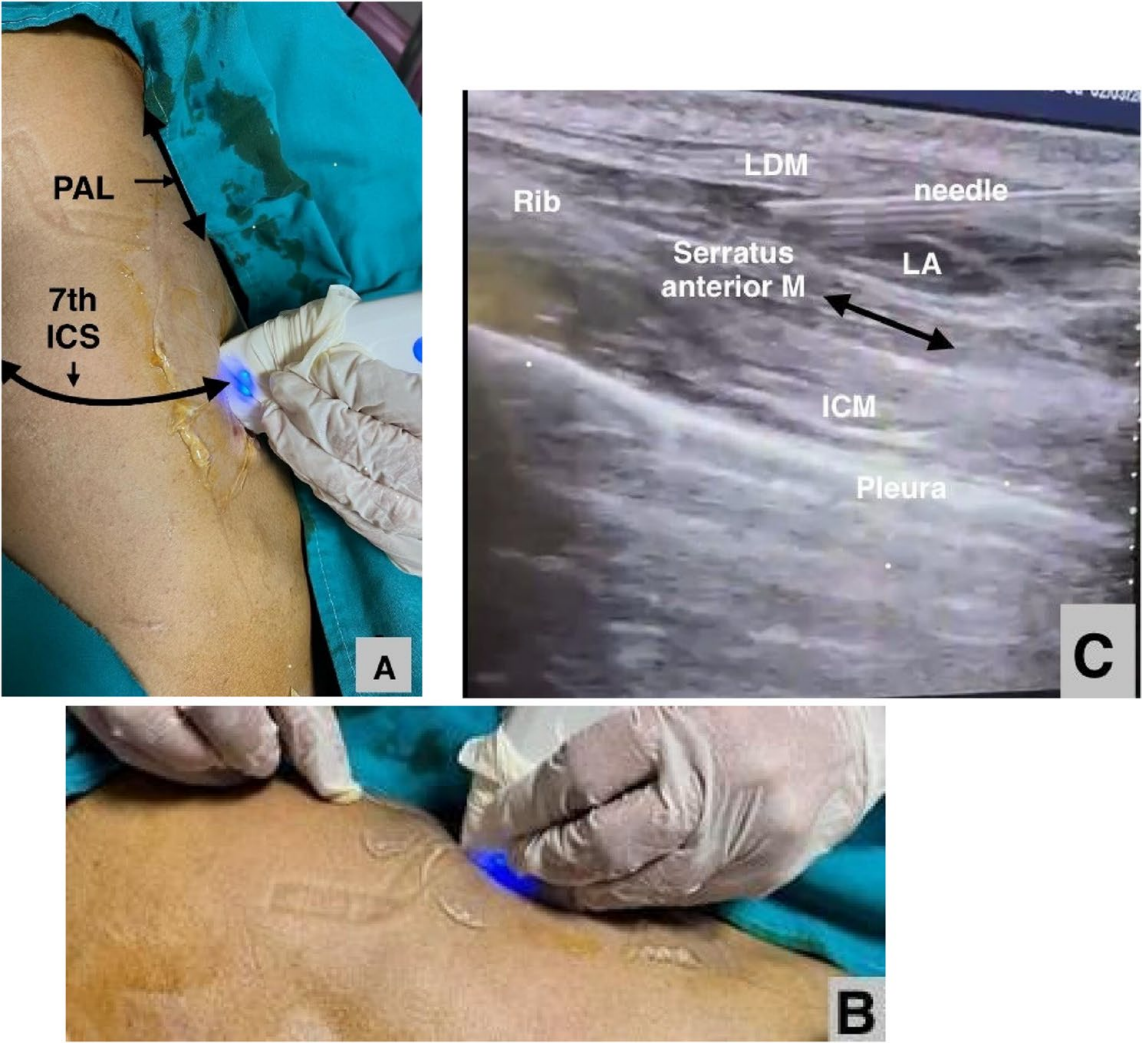
(**A**) Patients in lateral decubitus, the straight arrow represents posterior axillary line (PAL) and the linear probe held in longitudinal plane overlying the 7^th^ intercostal space (7^th^ ICS) (**B**) The block needle inserted in craniocaudal direction (**C**)Sonographic image of the modified serratus anterior plane block. LD: latissimus dorsi muscle, ICM: intercostal muscle, LA: local anesthetic spread between the serratus, and LD muscles

- ▪The technique of the MSAP block is as follows [14]:

Patients were positioned in the lateral decubitus position according to the selected site of surgical intervention. After sterilization and draping the area, a high-frequency linear ultrasound probe was placed obliquely on the second rib, and the probe was moved downwards laterally to the sixth or seventh rib in the posterior axillary line. The LD and SA muscles could be identified via ultrasound. A 22-gauge, 80 mm needle was inserted in-plane with the ultrasound probe and moved craniocaudally until it reached the interfacial plane of the SA and LD muscles. Negative aspiration confirmed the absence of blood, and then, 1 ml of normal saline was injected to create a hydrodissection sign and verify the needle tip. Finally, 25 ml of 0.25% bupivacaine was injected (Fig. 2 A-C).

In the two block groups, a 22-G needle was used every five minutes following LA injection, and the block was confirmed by the loss of pinprick sensation compared with the contralateral hemithorax. If at least six dermatomes from T2–T9 of the anterolateral hemithorax were covered, the block was deemed successful. The block was deemed unsuccessful, and the patient was excluded from the study if the desired sensory block level was not achieved after 30 min of LA injection. A block assessment was performed by an assistant not involved in the block performance or subsequent study steps. The study was designed as single-blind; the patient was aware of the group assignments, whereas the data collectors remained blind.

### Anaesthesia application:

Preoxygenation was carried out for 5 min with 100% O_2_ (4 L/min of O_2_). General anaesthesia was induced via the administration of 1 µg/kg fentanyl and 1–2 mg/kg propofol. Intubation was facilitated with 0.6 mg/kg rocuronium via a left double-lumen endotracheal tube of appropriate size (37–41 Fr) to allow deflation of the lung at the operative site. The capnograph was attached, and anaesthesia was maintained with sevoflurane (2–3%) supplemented with an O_2_ and air mixture. The lung was ventilated via volume-controlled ventilation adjusted to maintain the end-tidal carbon dioxide concentration (EtCO_2_) at 35–45 mmHg. To maintain adequate muscle relaxation, 0.15 mg/kg rocuronium was administered as needed. Intraoperative fentanyl was given as an additional bolus dose of 1 µg/kg with an increase in mean arterial blood pressure (MAP) or heart rate (HR) greater than 20% from baseline values.

### Surgical technique:

Three triangular incisions were used as part of the standard multiportal VATS technique to help with scoping and instrument insertion. Ports were placed at the 7th intercostal space mid-axillary line, 4th intercostal space anterior axillary line and the last port just anterior to the tip of the scapula. Assessment was performed via video thoracoscopy. Other surgical steps were performed according to each single surgical lesion. After completion of the procedure, one chest tube was inserted in the 7th intercostal space mid-axillary line. The muscles, soft tissues and skin were closed in anatomical fashion.

Thirty minutes before the end of the surgery, all patients, irrespective of their group assignments, received 1 gm of intravenous acetaminophen for postoperative pain management.

At the end of the operation, the patient received a 4 mg/kg dose of sugammadex to reverse the effect of the muscle relaxant. Once adequate muscle strength returned, the patient was extubated and transferred to the postanaesthesia care unit (PACU) with continued oxygen therapy and monitoring.

### **Postoperative:**

Standard postoperative analgesia in the form of a one-gram intravenous paracetamol infusion was administered four times a day (max dose 4 g/day). Rescue analgesia in the form of 50 mg of intravenous tramadol, with a maximum dose of 100 mg/6 hours, was given once the VAS score was ≥ 3.

## Primary outcome:

Twenty-four hours postoperative total tramadol consumption.

### Secondary outcomes:

- ▪The block performance time (min) was recorded from the time of placement of the ultrasound probe on the patient’s skin to the end of LA injection.
- ▪The time to first rescue analgesia was detected from the end of the surgery until the patient recorded a VAS score ≥ 3.
- ▪Pain intensity was assessed by the VAS. Pain was assessed at rest and during cough at 2, 4, 6, 8, 12, 16 and 24 h postoperatively.
- ▪Respiratory function: FVC and FEV1 were measured preoperatively and postoperatively at 4, 8, 12 and 24 h.
- ▪HR (beat/minute) and MAP (mmHg) were measured preoperatively (baseline); intraoperatively at 10, 20, 30, 40, 60, 80, 100,120 and 140 min; at the PACU and at 2 h postoperatively.
- ▪The anticipated side effects include LAs toxicity, nausea, vomiting, headache, constipation, dizziness and pulmonary complications.
- ▪Patient satisfaction with analgesia in the first 24 h after the operation was assessed via a 5-point Likert scale [17], where 5 represents very satisfied and 0 represents very dissatisfied.

### **Sample size:**

The sample size was calculated via Stata statistical software (Release 17). (StataCorP LLC. College Station, TX, 2021), considering the effect of ultrasound-guided MSAP block versus ultrasound-guided CSAP block on postoperative tramadol consumption in patients undergoing VATS.

According to a published study by Öksüz & Sayan [18], the total mean (± SD) analgesic drug (tramadol) consumption after 24 h in the bilateral CSAP block group was 120.4 (± 60.6), that in the unilateral CSAP block group was 156.42 (± 47.16), and that in the non-SAP block (control) group was 213.0 (± 26.2).

The null hypothesis was that there would be no difference among the studied groups. Assuming a large effect size (f = 0.4) and three groups, a one-way ANOVA F test for group effects was used to calculate the required sample size. With an alpha error of 5% and a power of 80%, a minimum sample size of 33 subjects per group (total 99 subjects) was determined.

### Statistical analysis and data interpretation:

Data were fed to the computer and analysed using IBM SPSS software package version 26 (IBM SPSS Statistics for Windows, Version 26.0. Armonk, NY: IBM Corp). After normality was tested via the Kolmogorov‒Smirnov test. Qualitative data were described using number and percent. The quantitative data were presented as median (minimum and maximum) for non-parametric data and mean, standard deviation for parametric data. Qualitative data were analysed via Chi-Square test for comparison of 2 or more groups. Fischer Exact test was used as correction for Chi-Square test when more than 25% of cells have count less than 5 in 2*2tables. Quantitative data were analysed via one-way ANOVA for parametric data and the Kruskal‒Wallis test for nonparametric data. A post hoc test (Tukey-HSD) was used after significant ANOVA, whereas a Bonferroni post hoc correction was used after a significant Kruskal‒Wallis test. In all the applied tests, p value < 0.05 was considered significant, p value ≥ 0.05 was considered nonsignificant.

## Results

**Fig. 3:**
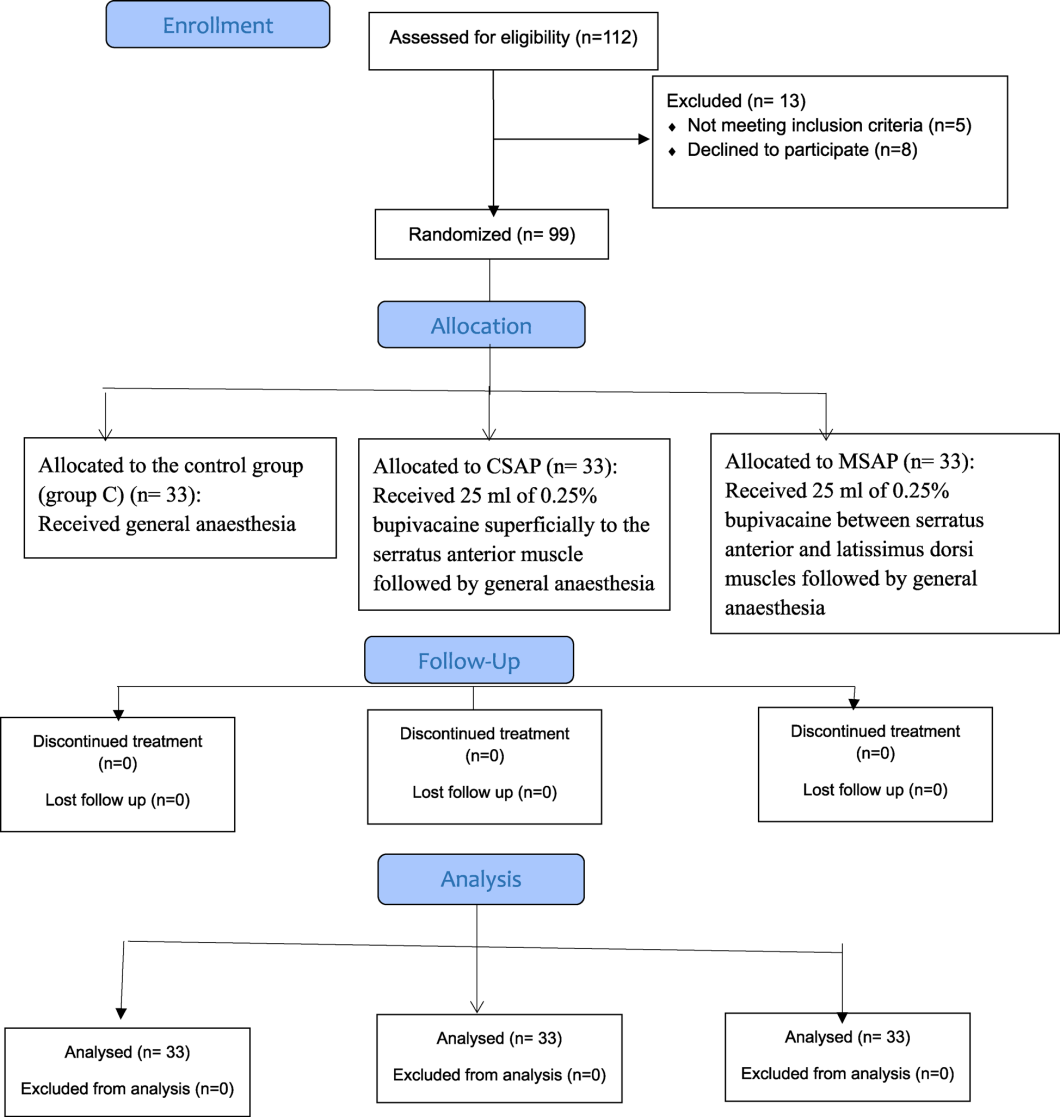
study flow chart (CONSORT diagram)

A total of 112 patients were assessed for eligibility; thirteen patients were excluded from the study, eight patients refused to participate, and five patients did not meet the inclusion criteria (two patients had ischaemic heart disease, two had advanced liver disease, and one was using opioids chronically). The remaining 99 patients were enrolled and randomly assigned to 3 equal groups (33 patients each) (Fig. 3).

Patient characteristics were not significantly different between the groups. Similarly, there was no significant difference between the groups in terms of the operative data (Table 1).

**Table 1:** Analysis of patient's characteristics and operative data in the studied groups:

		**C Group ** **(n= 33)**	**CSAP group** **(n= 33)**	**MSAP group** **(n= 33)**	**P value **
Age (years)		46.21 ± 13.53	44.58 ± 13.49	43 ± 12.91	0.620
Gender	**Male**	21 (63.6%)	16 (48.5%)	14 (42.4%)	0.458
	**Female**	12 (36.4%)	17 (51.5%)	14 (42.4%)	
BMI (Kg/m^2^)		27.07 ± 3.02	28.20 ± 2.70	26.65 ± 2.63	0.071
ASA	**ASA I**	9 (27.3%)	12 (36.4%)	11 (33.3%)	0.724
	**ASA II**	24 (72.7 %)	21(63.6 %)	22 (66.7 %)	
Type of operation	**Lobectomy**	7 (21.2%)	10 (30.3%)	7 (21.2%)	0.779
	**Segmentectomy**	15 (45.5%)	16 (48.5%)	17 (51.5%)	
	**Wedge resection**	11 (33.3%)	7 (21.2%)	9 (27.3%)	
Block performance (min)		------------	8.45 ± 1.50	8.36 ± 1.25	0.790
Operative time (min)		119.09 ± 15.69	118.94 ± 15.45	115.15 ± 14.97	0.501

Quantitative data are expressed as mean ± SD (Data were compared using one-way ANOVA test)

Qualitative data are expressed as number (percent) (Data were compared using Chi-square test)

*C* group: control group, *CSAP* group Conventional serratus anterior block group, *MSAP* group Modified serratus anterior block group, *BMI* Body mass index

The total tramadol dose at 24 h was the lowest in the MSAP group and the highest in the control group (p value < 0.001). Moreover, the time to first rescue analgesia was significantly longer in the MSAP group than in the CSAP group and the control group (p value < 0.001) (Table 2).

**Table 2:** Post-operative analgesic data in the studied group

	C Group (n= 33)	CSAP group (n= 33)	MSAP group (n= 33)	P value	multiple groups comparisons	95% CI
Postoperative Total Tramadol dose (24 hours) (mg)	301.58±34.61	200 ± 26.53	130.18±23.26	< 0.001*	p1< 0.001* p2 < 0.001* P3 < 0.001*	**- 160.62: −241.46** **−206.63: -273.80** **- 44.33: - 105.76**
Time of postoperative first analgesic recall (min)	42.90 ± 9.61	380.5 ± 33.55	475.40±21.55	< 0.001*	p1< 0.001* p2 < 0.001* P3 < 0.001*	**88.32: 116.63** **170.07: 198.52** **57.15: 79.12**

Data were expressed as mean and standard deviation and were compared using one-way ANOVA test

* C* group Control group, *CSAP* group Conventional serratus anterior block group, *MSAP* group Modified serratus anterior block group

*indicate significant difference

P1: Significance between group C and CSAP group

P2: Significance between group C and MSAP group

P3: Significance between group CSAP and MSAP group

The visual analogue scores followed a similar pattern at rest and during cough. At 2, 4 and 6 h postoperatively, the VAS scores were significantly greater in the control group than in the other two groups but were comparable between the CSAP and MSAP groups (p values: 0.002,** <** 0.001 &** < **0.001). At 8 postoperatively, the VAS score was highest in the control group and lowest in the MSAP group (P value was **< **0.001**)**. However, at 12, 16, and 24 h, the VAS scores were comparable among the three groups (Table 3).

**Table 3:** Analysis of visual analogue scale (VAS) score at rest and during cough in the studied groups:

	**Group C** **(n= 33)**	**CSAP group** **(n= 33)**	**MSAP group** **(n= 33)**	**p**	**multiple groups** **comparisons**	**95% CI **
Analysis of VAS score at rest in the study groups						
2 hours	2 (1–2)	1 (0 - 1)	1 (0 - 1)	0.002*	p1= 0.005* p2 = 0.005* p3 = 0.990	**0.79: 1.39** **0.88: 1.48 ** −0.21: 0.39
4 hours	3 (3 - 5)	1 (1–2)	1 (0 - 1)	< 0.001*	p1< 0.001* p2 < 0.001* p3 = 0.642	**1.48: 2.22** **2.24: 2.97** 0.39: 1.12
6 hours	4 (4 - 5)	2 (1–2)	2 (0 - 2)	< 0.001*	p1< 0.001* p2 < 0.001* p3 = 0.279	**2.38: 3.08** **2.62: 3.32** −0.11: 0.59
8 hours	4 (3 - 5)	3 (2- 3)	2 (1 - 2)	< 0.001*	p1< 0.001* p2 < 0.001* p3 = 0.001*	**0.90: 1.64** **1.90: 2.64** **0.63: 1.37**
12 hours	4 (4 - 5)	4 (3- 5)	4 (3 - 4)	0.094	p1= 0.276 p2 = 0.586 p3 = 0.312	0.34: 1.06 0.49: 1.21 −1.06: −0.34
16 hours	4 (3 - 5)	4 (3 - 5)	4 (3 - 5)	0.358	p1= 0.782 p2 = 0.750 p3 = 0.815	−0.63: 0.33 −0.54: 0.42 −0.39: 0.57
24 hours	4 (3 - 5)	4 (3 - 4)	4 (3 - 4)	0.438	p1= 0.782 p2 = 0.750 p3 = 0.815	0.15: 0.82 0.06: 0.73 −0.42: 0.24
General linear model	< 0.001*	< 0.001*	< 0.001*			
Analysis of VAS score during cough in the study groups						
2 hours	2 (1–2)	1 (0 - 1)	1 (0 - 1)	0.001*	p1= 0.004* p2 = 0.003* p3 = 0.984	**0.83 :1.41** **0.86: 1.44** −0.26: 0.32
4 hours	3 (3 - 5)	2 (2–3)	2 (1 - 2)	< 0.001*	p1< 0.001* p2 < 0.001* p3 = 0.360	**0.75: 1.49** **1.51: 2.25** 0.39: 1.12
6 hours	4 (4 - 5)	2 (2–3)	2 (1 - 2)	< 0.001*	p1< 0.001* p2 < 0.001* p3 = 0.282	**1.43: 2.02** **2.43: 3.02** 0.71: 1.29
8 hours	4 (4 - 5)	4 (4- 5)	4 (3 - 4)	< 0.001*	p1< 0.001* p2 < 0.001* p3 = 0.001*	**0.18: 0.73** **0.75: 1.31** **0.30: 0.85**
12 hours	4 (4 - 5)	4 (3- 5)	4 (3 - 5)	0.112	p1= 0.302 p2 = 0.564 p3 = 0.725	0.32: 1.08 0.29: 1.05 −0.41: 0.35
16 hours	4 (4 - 6)	4 (3 - 6)	4 (3 - 5)	0.258	p1= 0.650 p2 = 0.412 p3 = 0.572	0.66: 1.65 0.23: 1.22 −0.92: 0.07
24 hours	5 (3 - 5)	4 (3 - 5)	4 (3 - 4)	0.360	p1= 0.724 p2 = 0.548 p3 = 0.778	0.00: 0.73 0.03: 0.76 −0.33: 0.39
General linear model	< 0.001*	< 0.001*	< 0.001*			

Data were expressed as median and range and were compared using Kruskal Wallis test

*C* group Control group, *CSAP* group Conventional serratus anterior block group, *MSAP* group Modified serratus anterior block group

*indicate significant difference

P1: Significance between group C and CSAP group

P2: Significance between group C and MSAP group

P3: Significance between group CSAP and MSAP group

When respiratory function (FVC, FEV1) was compared, the preoperative values were comparable among the three groups. Compared with the preoperative value, there was a statistically significant reduction in respiratory function at all time points. At 4 h after surgery, the significantly highest value was in the MSAP group, whereas the lowest value was in the control group. At 8 h, the MSAP group presented a significantly greater value than did the other groups, with no significant difference between the CSAP group and the control groups. At 12 and 24 h, the values were comparable among the three groups. (Table 4).

**Table 4:** Analysis of pulmonary function test (forced vital capacity and forced expiratory volume in one second) (%) in the studied groups:

	**Group C** **(n= 33)**	**CSAP group** **(n= 33)**	**MSAP group** **(n= 33)**	**p**	**multiple groups** **comparisons**	**95% CI **
Analysis of forced vital capacity (FVC) (%) in the study groups						
Preoperative	77.52 ± 1.03	78.48 ± 0.91 A	78.55 ± 0.94	0.426	p1= 0.842 p1= 0.869 p1= 0.926	−1.45: - 0.49 −1.52: −0.54 −0.51: 0.39
4 hours	49.18 ± 2.31	62.15 ± 1.23	68.27 ± 1.57	< 0.001*	p1< 0.001* p2 < 0.001* P3 = 0.012*	**−13.88: −12.06** **−20.06: −18.12** **−6.81: −5.43**
Paired sample t-test (with baseline)	< 0.001*	< 0.001*	< 0.001*			
8 hours	62.67 ± 1.55	64.52 ± 2.92	69.27 ± 1.57	< 0.001*	p1= 0.597 p2 = 0.013* P3 = 0.040*	−3: −0.70 **−7.37: −5.84** **−5.91: −3.61**
Paired sample t-test (with baseline)	< 0.001*	< 0.001*	< 0.001*			
12 hours	62.76 ± 1.46	64.82 ± 3.12	65.27 ± 1.57	0.285	p1= 0.592 p2= 0.506 p3= 0.714	−3.26: −0.86 −3.26: −1.77 −1.67: 0.76
Paired sample t-test (with baseline)	< 0.001*	< 0.001*	< 0.001*			
24 hours	67.61 ± 1.71	67.88 ± 1.43	67.52 ± 1.60	0.748	p1= 0.910 p2= 0.924 p3= 0.902	−1.05: 0.50 −0.72: 0.91 −0.38: 1.11
Paired sample t-test (with baseline)	< 0.001*	< 0.001*	< 0.001*			
General linear model	< 0.001*	< 0.001*	< 0.001*			
Analysis of forced expiratory volume in one second (VEF1)						
Preoperative	76.45 ± 0.83	77.97 ± 1.05	77.55 ± 1.20	0.408	p1= 0.877 p2= 0.896 p3= 0.934	−1.98: −1.05 −1.60: −0.58 −0.13: 0.98
4 hours	49.36 ± 2.51	63 ± 1.58	67.55 ± 1.33	< 0.001*	p1< 0.001* p2 < 0.001* p3 = 0.015*	**−14.67: −12.60** **−19.17: −17.19** **−5.26: −3.83**
Paired sample t-test (with baseline)	< 0.001*	< 0.001*	< 0.001*			
8 hours	52.24 ± 1.25	53.88 ± 2.93	67.79 ± 1.82	< 0.001*	p1= 0.885 p2< 0.001* p3 < 0.001*	−2.75: −0.53 **−16.31: −14.78** **−15.11: −12.71**
Paired sample t-test (with baseline)	< 0.001*	< 0.001*	< 0.001*			
12 hours	58.58 ± 1.20	59.58 ± 3.12	59.73 ± 1.68	0.327	p1= 0.826 p2= 0.820 p3= 0.904	−2.16: 0.16 −1.87: −0.43 −1.39: 1.08
Paired sample t-test (with baseline)	< 0.001*	< 0.001*	< 0.001*			
24 hours	67.64 ± 1.27	67.61 ± 1.80	67.76 ± 1.58	0.736	p1= 0.996 p2= 0.975 p3= 0.962	−0.74: 0.80 −0.83: 0.58 - 0.99: 0.68
Paired sample t-test (with baseline)	< 0.001*	< 0.001*	< 0.001*			
General linear model	< 0.001*	< 0.001*	< 0.001*			

Data were expressed as mean and standard deviation were compared using one-way ANOVA test)

*C* Group Control group, *CSAP* group Conventional serratus anterior block group, *MSAP* group Modified serratus anterior block group

*indicate significant difference

P1: Significance between group C and CSAP group

P2: Significance between group C and MSAP group

P3: Significance between group CSAP and MSAP group

**Fig. 4:**
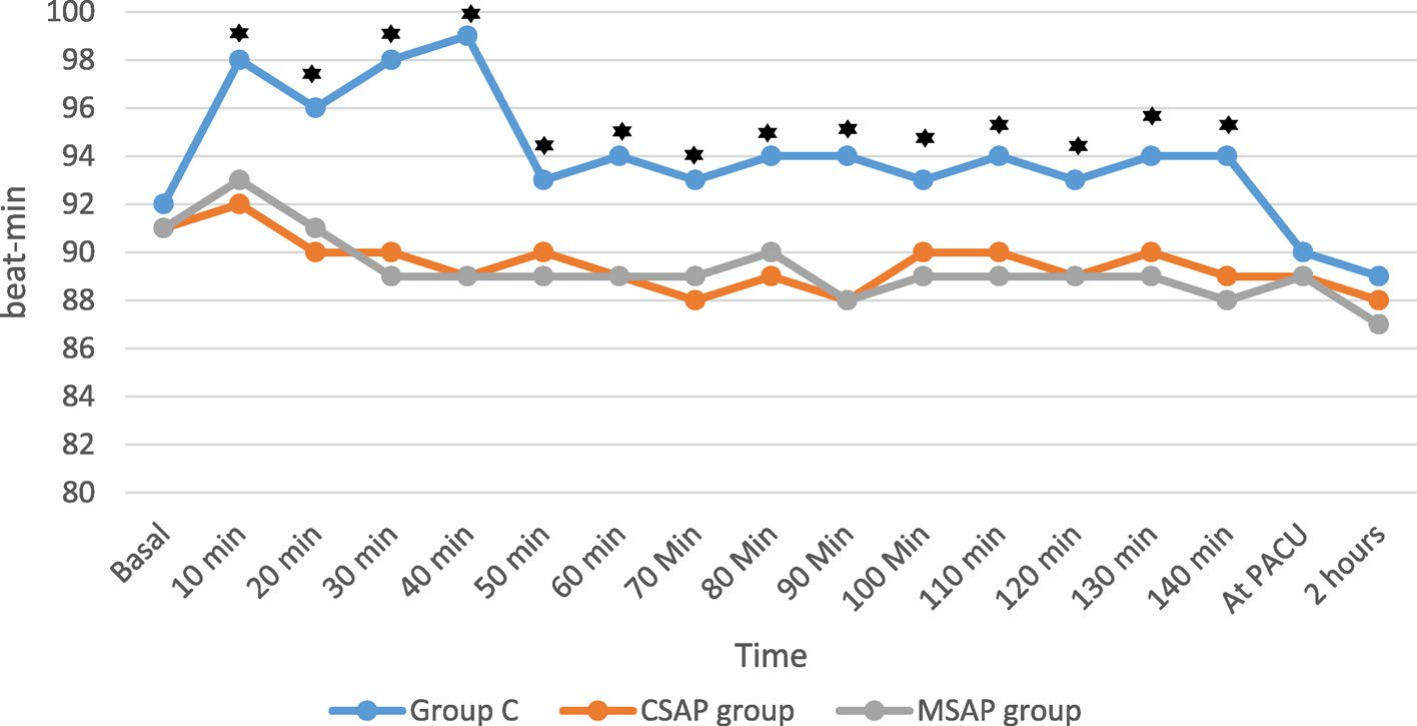
Perioperative heart rates of the studied groups

The basal heart rate values were comparable among the three groups. However, HR values were greater in the control group and were comparable in the two block groups at all intraoperative time points. At the PACU and at 2 h postoperatively, the HR values were not significantly different between the three groups (Fig. 4).

**Fig. 5:**
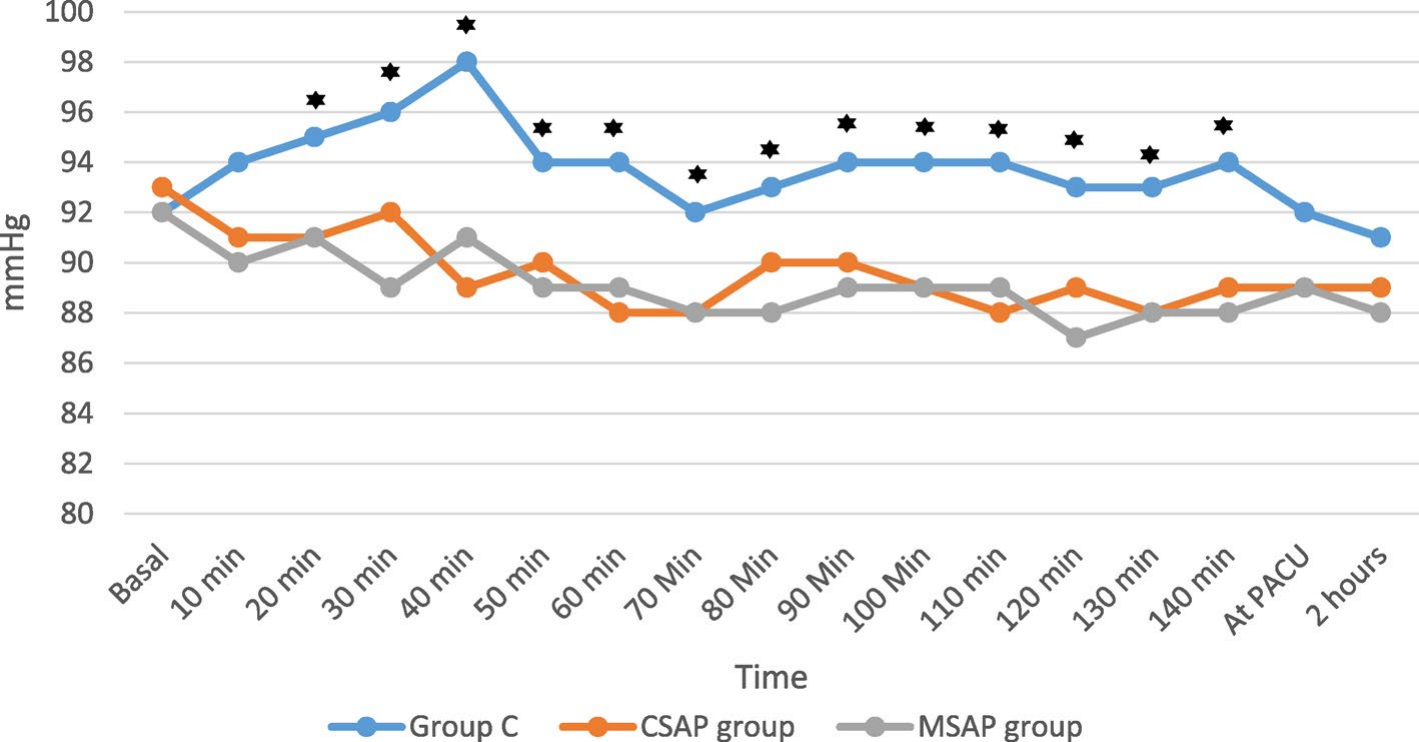
Perioperative mean blood pressure (MAP) of the studied groups

Blood pressure values were not significantly different at baseline or at 10 min intraoperatively. However, blood pressure values were significantly greater in the control group and comparable between the two block groups at 20, 30, 40, 60, 80, 100, 120 and 140 min intraoperatively. At the PACU and at 2 h postoperatively, blood pressure values were comparable among the three groups (Fig. 5).

Our results also revealed a greater incidence of nausea, vomiting and constipation in the control group than in the other two groups; furthermore, lower satisfaction was reported in the control group than in the other two groups (Table 5).

**Table 5:** Analysis of complications and patient’s satisfaction in the studied groups:

**Variables **	**Group C** **(n= 33)**	**CSAP group** **(n= 33)**	**MSAP group** **(n= 33)**	**p**	**multiple groups** **comparisons**
LA toxicity	0 (0%)	0 (0%)	0 (0%)	_____	
Nausea	9 (27.3%)	3 (9.1%)	2 (6.1%)	0.028*	p1 = 0.032* p2= 0.021* p3 = 0.952
Vomiting	6 (18.2%)	1 (3%)	1 (3%)	0.033*	p1 = 0.046* p2= 0.046* p3 = 1
Headache	5 (15.2 %)	3 (9.1%)	0 (0%)	00075	
Constipation	6 (18.2%)	1 (3%)	1 (3%)	0.033*	p1 = 0.046* p2= 0.046* p3 = 1
Dizziness	0 (0%)	0 (0%)	0 (0%)	_____	
Pulmonary complications	2 (6.1%)	0 (0%)	0 (0%)	0.130	
Analysis of patients’ satisfaction in the study groups					
Very dissatisfied	10 (30.3%)	0 (0%)	0 (0%)	< 0.001*	
Dissatisfied	21 (63.6%)	0 (0%)	0 (0%)		
Neutral	2 (6.1%)	6 (18.2%)	0 (0%)		
Satisfied	0 (0%)	19 (57.6%)	21 (63.6%)		
Very satisfied	0 (0%)	8 (24.2%)	12 (36.4%)		

Data were expressed as number and percent and were compared using Chi-square/Monte-carlo test)

*C* group Control group, *CSAP* group Conventional serratus anterior block group, *MSAP* group: Modified serratus anterior block group

*indicate significant difference

P1: Significance between group C and CSAP group

P2: Significance between group C and MSAP group

P3: Significance between group CSAP and MSAP group

## Discussion

Although VATS is a minimally invasive technique and is preferable to open surgery for preserving pulmonary function [19–21], it can lead to significant morbidity due to distractive and compressive effects of torquing, multiple ports, and the force used to retract the ribs, which may cause nerve injury [22]. Approximately 85% of patients experience moderate to severe post-VATS pain during coughing or moving [23], and nearly 22–63% suffer from chronic pain [24]. Optimal pain control can ensure adequate breathing, enforce coughing, prevent lung atelectasis and accelerate respiratory function recovery [25]. For VATS, surgical analgesia was required throughout the anterior, lateral, and partially posterior walls of the ipsilateral chest.

Conventional serratus anterior plane block is a promising technique that has demonstrated beneficial effects on analgesia and recovery in VATS [26]. By blocking mainly the lateral cutaneous branches of the intercostal nerves feeding the anterolateral chest wall and the long thoracic nerve supplying the SA muscle, CSAP produces an analgesic effect [11, 13]. In the present study, the CSAP block was performed superficially to the SA muscle, as superficial CSAP has the advantages of simple performance and a wider range of dermatomal blocks with a longer duration of action and is not affected by obesity or the use of anticoagulants [11, 27–29].

The modified serratus anterior plane block technique, in which the ultrasound probe is positioned even more caudally and posteriorly toward the posterior axillary line, has the same mechanism of action as CSAP, which blocks lateral cutaneous branches of the intercostal nerves and the long thoracic nerve. It additionally blocks the thoracodorsal nerve, the sole nerve that supplies the LD, and creates a plexus between the LD and SA muscles [14]. This may help to extend the analgesic effect of this block to a portion of the posterior chest wall covered by latissimus dorsi that may contain VATS ports. Accordingly, and given the lack of randomized clinical trials comparing the analgesic effect of MSAP block with that of CSAP block in VATS surgeries. The authors decided to conduct a randomized control trial comparing the two approaches.

In the present study, the control group had the significantly highest 24-h tramadol consumption and VAS scores during the first 8 h postoperatively. Additionally, the time of first-rescue analgesia was significantly shorter than that in the two-block groups. Furthermore, when the two blocks were compared, the MSAP block had significantly lower tramadol consumption and a longer analgesic duration. The VAS scores were comparable between the two groups at different postoperative time points, except at 8 h, when the score was lower in the MSAP group than in the CSAP group. This may be explained by the fact that MSAP, as it performs more posteriorly, additionally targets the thoracodorsal nerve and forms a plexus between the LD and SA muscles [14], which could be beneficial in VATS since its two ports are passed through the LD muscle; one is inserted through the 7th intercostal space at the mid-axillary line, and the other is anterior to the inferior angle of the scapula, which may contribute to alleviating the resulting pain; moreover, the plexus located under the pectoralis muscle is also blocked [14, 30].

These results are consistent with those of Khemka and Chakraborty [14], who reported in their case series of MSAP blocks in breast oncoplastic surgery that all patients remained pain-free for up to 9 h after surgery. The patients were given 3 mg of morphine intravenously when the VAS score was greater than 3, and none of them needed more morphine through the PCA.

Li et al**.**, in a meta-analysis, demonstrated that single-shot CSAP block can be effective in alleviating postoperative pain and decreasing postoperative opioid consumption in patients undergoing VATS [31]. Moreover, CSAP block has been reported to be not inferior to PVB or TEB in VATS [13, 32].

The 2019 guidelines from the European Society of Thoracic Surgery [33] emphasize the crucial role of pain management, often achieved through locoregional anaesthesia, in facilitating enhanced recovery after surgery [34] and decreasing postoperative complications such as hypoxia, atelectasis and pneumonia [35].

Therefore, another point of interest in the current study is the follow-up of changes in pulmonary function tests after surgery, where a substantial early postoperative reduction in pulmonary function (FVC and FEV1) was reported in all three groups compared with the preoperative values, and this reduction was significantly greater in the control group. The best values of pulmonary function tests were found in the MSAP group during the first postoperative 8 h. This is attributed to the best pain management in this group, which allowed patients to perform effective breathing exercises for adequate gas exchange.

In accordance with these results, Gao et al. reported that continuous CSAP block led to better lung function rehabilitation in patients undergoing VATS, as indicated by greater increases in FEV_1_, FVC, and FEV_1_/FVC in the continuous CSAP group than in the patient-controlled intravenous analgesia (PCIA) group, and the incidence of complications, such as atelectasis, pneumonia, and hypoxemia, was significantly reduced [36]. Additionally, Hernandez et al. concluded that incentive spirometry volumes improved significantly after the CSAP block in patients with multiple rib fractures [37].

With respect to hemodynamics, HR and MAP in the present study were significantly greater in the control group than in the two block groups, with no significant difference between the block groups at all intraoperative time points. This is because CSAP and, by extension, MSAP blocks can effectively maintain hemodynamic stability, as they block the superficial nerves in the anterior and lateral chest wall without causing sympathetic block or hypotension [38].

The current results are consistent with those of Zhang et al.‘s study, where CSAP block significantly stabilized perioperative vital signs in patients undergoing thoracoscopic surgery [39].

In terms of postoperative complications, the incidence of nausea, vomiting and constipation was significantly greater in the control group than in the other two blocks groups. These findings are in accordance with the findings of a previous meta-analysis, which concluded that CSAP significantly decreased the occurrence of postoperative nausea and vomiting (PONV) [40].

The authors’ experience revealed that MSAP block is easier to perform than CSAP block and provides a better view of the ribs and muscle plane, especially in obese women with large breasts. Furthermore, patient satisfaction, which is a useful tool for evaluating health services and patient care and is considered the cornerstone of healthcare quality management [41, 42], revealed that the highest degree of satisfaction was found in the MSAP. This can be attributed to better pain management, greater comfort during coughing, as reflected by higher lung function test results, stable hemodynamics, and a reduced incidence of constipation, nausea and vomiting.

The limitations of this study are that it was a single- center study and was limited to certain groups of patients, which might limit the generalizability of these findings; therefore, multicenter studies and potential confounding variables such as patient comorbidities are needed to validate the results. The short-term follow-up of the outcomes may not provide a comprehensive overview of the long-term benefits for chronic pain or complications. Additionally, patient blindness could not be assured, as the block was performed before the induction of general anaesthesia, and the patients were awake and oriented to the block position. In addition, the scarcity of clinical studies related to MSAP block made it difficult to compare these results with similar results, but we tried to address this deficit by comparing the results with those of its counterpart, CSAP block; therefore, further studies may also be needed to compare this modified approach with other regional blocks and to investigate the effects of other local anaesthetic and adjuvant drugs with this approach.

##  Conclusion

Compared with the other two groups, the modified serratus anterior plane block group presented the lowest tramadol consumption, longest analgesic time, best pulmonary function recovery and stable hemodynamics.

## Supplementary Information

Supplementary Material 1.

## Data Availability

The corresponding author can provide the data utilized and analyzed in this study upon reasonable request.
