# Supplementary material for: Ultrasound-guided modified versus conventional serratus anterior plane block as a preemptive analgesic for unilateral video-assisted thoracoscopic surgery
Source: BMC Anesthesiol. 2025 Aug 29;25:435. doi: 10.1186/s12871-025-03314-5 (PMC12395965; doi:10.1186/s12871-025-03314-5)
Supplement: Supplementary file 1 — Supplementary Material 1. [file 12871_2025_3314_MOESM1_ESM.docx]

**Declarations**

**Ethics approval and consent to participate.**

This prospective randomized controlled clinical study was carried out on elderly patients scheduled for unilateral video assisted thoracoscopic surgery at Zagazig University hospitals. The ethical approval from the institutional review board (The research ethical committee of Faculty of Medicine, Zagazig University) was obtained with the reference number (IRB #10060/30-10-2022). This study was registered under clinicaltrials.gov (NCT05661253). Written informed consent was obtained from all participants after they understood the concept of this research. The study was carried out in accordance with the guidelines and regulations of the Helsinki Declarations.

**Availability of data and materials**

The data used and analyzed during our study are available from the corresponding author on reasonable request.

**Competing interests**

The authors have no competing interests.

**Funding**

This research did not receive any specific grant from funding agencies in the public, commercial, or not-for-profit sectors. Open access funding provided by The Science, Technology & Innovation Funding Authority (STDF) in cooperation with The Egyptian Knowledge Bank (EKB).

**Consent for publication**

Not applicable.

**Authors’ contributions**

All authors reviewed the final manuscript and approved it. Shereen E. Abd Ellatif and Heba M. Fathi registered, collected, and analyzed the data, helped within the design of the study, and wrote the main manuscript text. Asmaa M. Galal Eldin prepared the tables and figures, helped with the study design, and writing review and editing. Ehab Sabry Ali supervision, analyzed the data, and helped with the study design.

**Corresponding author**

Correspondence to Shereen E. Abd Ellatif

**Writing process:**

Authors did not use AI in the writing process of this research.

**Acknowledgments**

The authors acknowledge the Faculty of Human Medicine staff, Zagazig University Hospitals, who facilitate the measurements.
